# Deviations of the Nasal Septum

**Published:** 1891-06-27

**Authors:** 


### RHINOLOGY.

### DEVIATIONS OF THE NASAL SEPTUM.

Deviations of the nasal septum have long been known as a very common anatomical condition, but their clinical import has only comparatively recently been recognised. Few of us can ever have conceived the very numerous and serious pathological conditions the responsibility for which rhinologists have endeavoured to fasten on to the nasal septum; and yet, although too much importance seems to have been attached to co-existent septal deviations in some cases, there is now no doubt whatever that these deviations are truly the *fons et origo mali* in many others. Only a small percentage of adults possess a straight septum, and as only a very small percentage of adults suffer from the effects of their deviated septa, it follows that the deviation *per se* is not answerable for the pathological symptoms arising in the few. The conditions in which treatment of the deviation is called for have been well summarised by Drs. Moore and Bergonié, viz.: (1) Stenosis of one or both nasal fossæ, impeding respiration from being properly performed by these cavities, and putting the entire organism under conditions of defective vitality. We all know too well the *rôle* of the nose in the physiological act of respiration to underrate the troubles which result from obstruction of these air channels, and consequently the formal indication for limiting this state of things within the limits of possibility. Nasal stenoses, whether congenital or acquired, have, moreover, the inconvenience of very often causing mischievous effect upon the organ of hearing, either by causing a nasal catarrh of which the propagation to the Eustachian tube or middle ear is very fatal to this organ, or in causing complications of tubal affections or pre-existent affections of the tympanum. (2) Reflex neuroses. When the outgrowths, spines, or asymmetries of the septum, without being large enough to impede respiration, become the point of departure for those nervous affections so various and so well known to-day (migraines, neuralgias, glottic spasms, spasmodic cough, &c.) it is necessary to remove the spine which causes so much trouble, or at least which acts as a determining cause. 3 Æsthetic. When the malformation is the result of a traumatism (blow or fall upon the nose) which has occasioned either a fracture of the septum and the formation of a vicious callus, or an enchondroma or exostosis consecutive to a perichondritis or periostitis; when a depression of the nasal skeleton, or a considerable deformity of this organ results, we may then, in certain cases and subjects, interfere surgically if we may hope to remedy this state of things, and to obtain for the patient a sufficiently proper nose.

As regards the method of operating in the cases indicated under these three headings, there is (*a*) treatment of deviations or crests. When the deviation is simple and relatively easy to correct, the finger may be employed, or the apparatus of Turasz or Delstanche. These apparatus, the form of which should always be adapted to each case, can only be really efficacious in adolescents and in the case of simple incurvation and recent luxations of the fibro-cartilage. The existence of spurs or sharp crests constitutes almost a contra-indication to these replacements, which are often difficult and always long to obtain. The most simple and rapid method of arriving at the result desired is still surgical treatment, which varies with each patient and with the end in view. If it is desired merely to render respiration through the nose more easy, we may, as recommended by Blandin, make a communication between the two nasal cavities by removing a piece of cartilage with the punch. Chassaignac dissected the pituitary membrane, and removed the deviated portion by resection; this is, indeed, the proceeding generally used at this time, the instruments for which have varied merely. The removal by the saw has many drawbacks. One of the disadvantages of the saw is the amount of hæmorrhage. If an anæsthetic is administered the hæmorrhage is very inconvenient to the operator, as well as to the anæsthetist, and if no anæsthetic be given the patient is apt to be nervous and restless.

Dr. Moure has long endeavoured to find a method without pain, and especially without hæmorrhage. Such a process was introduced in France and used by Dr. C. Miot, and by Drs. Garel and Moure. The results obtained have been so encouraging that Dr. Moure does not hesitate to give preference to this method of treating deviations (with or without thickenings), and of osseous or cartilaginous growths of the nasal septum, and this method is *electrolysis*. With it we can operate as energetically or as lightly as desired, and, thanks to cocaine, it is made almost painless; and a point of capital importance, the septum is destroyed without any flow of blood, or at least with scarcely a few drops which stain the handkerchief when the electrolysis is ended. Dr. Moure has employed this method for more than two years—he has resected a great number of abnormal septa, and the result has in all cases been all that he sought. There is no doubt that a considerable number of patients in whom, as the result of various thickening of the mucous membrane of the nares being superadded to the congenital septal deformity, partial or complete stenosis, with all the evil secondary results, may be completely relieved without any operation or any interference with the septum.*

* Journ. of Laryng., Mar., 1891; ibid., Dec., 1890.

